# Supplementary material for: Psychosocial experiences of adolescents with tuberculosis in Cape Town
Source: PLOS Glob Public Health. 2024 Sep 20;4(9):e0003539. doi: 10.1371/journal.pgph.0003539 (PMC11414892; doi:10.1371/journal.pgph.0003539)
Supplement: Attachment — Submitted filename: Response to Reviewers.docx [file pgph.0003539.s001.docx]

Dear Editorial Team,

We would like to thank the editorial team and the reviewers for their helpful comments and suggestions.

We have carefully considered each of the reviewers’ comments and have made changes to the manuscript in response.

## Response to Reviewer:

We would like to thank the editor and reviewers for their comments and suggestions. We agree with the reviewer that these additions will improve the analytical clarity of the manuscript. In the table below we include the reviewer’s comments (on the left) and our responses (on the right). For ease of reference, we have indicated where and what changes were made to the manuscript.

## Editor Comments

| **Reviewers’ comments** | **Response to reviewer and edits to manuscript** |
| --- | --- |
| **Journal Requirements:** Please review your reference list to ensure that it is complete and correct. If you have cited papers that have been retracted, please include the rationale for doing so in the manuscript text or remove these references and replace them with relevant current references. Any changes to the reference list should be mentioned in the rebuttal letter that accompanies your revised manuscript. If you need to cite a retracted article, indicate the article’s retracted status in the References list and also include a citation and full reference for the retraction notice. | Thank you for this comment. We have reviewed the reference list and confirm that it is complete and correct. |
| **Academic Editor Comments:** -The manuscript should be revised for punctuation and formatting errors.  -The beginning of the Discussion section can be made better. The first paragraph for example could offer a summary of the findings, and also explain how this study is different from the rest of the studies conducted on a similar subject. | Thank you. We have reworked the discussion section to bring it in line with your suggestions. See lines 385-397:  *This study sough to understand adolescents’ experiences of TB (both DS- and MDR-TB) in the context of their psychosocial, economic and household contexts. We found that TB negatively impacts adolescents’ everyday functioning, with some adolescents ceasing activities outside the household. Generally, adolescents experienced distress, isolation and tension in their romantic, platonic and familial relationships, preventing them from forming meaningful connections with others. As expected, environmental circumstances like unemployment and generalised insecurity was common among our participants and their families, hindering their ability to exercise their agency and make health-conscious decisions. TB disrupted adolescents schooling, raising concerns about the long-term impact on their financial freedom.* |

## Reviewer #1:

| 1. Please summarize the study findings as a table at the end of the results section. | 1. Thank you. We have summarised the study findings as per your suggestions. See Table 1 on page 13. |
| --- | --- |
| 2. Please avail the code book | 2. Thank you for the request. Apologies for the confusion. Since, we utilised Moscibrodski’s framework as our starting point, we did not develop a codebook from scratch (i.e., inductive coding). Instead, we used the framework to deductively analyse the data. Here the coding process refers to identifying insights and quotes from the participants’ case files in line with Moscibrodski’s existing themes. So, the ‘codebook’ developed from the case descriptive method, does not comprise direct quotes from every participant. Instead, it comprises researchers’ insights/summations of participants’ responses to the interview questions, organised around the thematic areas outlined in Moscibrodski’s framework. The summary of findings table provides an overview of responses from participants for each thematic area. (See Table 1 on page 13).   For the sake of clarity, we have removed reference to coding and the codebook from the methods section. In Lines 133-136, we have reworded ‘intercoder reliability’ to ‘implementation and analytic uniformity’, so that the sentence now reads:  *The graduate researchers (KM and SJ) received study-specific training on how to execute the participatory research activities to achieve implementation and analytic uniformity.*  Lines 152-153 have been reworded to say:  *The interpretation of data was checked by GH and JS for analytic uniformity.* |
| 3. Please avail a duly completed COREQ checklist as a supplementary. | 3. Thank you. The COREQ checklist has been completed and uploaded as supplementary material. |

## Response to Reviewer:

We would like to thank the reviewer for their comments and suggestions. In the table below we include the reviewer’s comments (on the left) and our responses (on the right). For ease of reference, we have indicated where and what changes were made to the manuscript.

## Reviewer #2:

An intriguing article that explores adolescents’ experiences of TB in Cape Town. I have two minor edits

| **Reviewers’ comments** | **Response to reviewer and edits to manuscript** |
| --- | --- |
| 1. Line 235 - You have 'A 17-year-old you man'. You need to edit this line for correctness and clarity. | 1. Thank you for bringing this to our attention. We have edited this sentence as follows (see line 237):   *“A 17-year-old boy conceded that TB affected how “I spend time with the rest of my loved ones."*  We took participants’ gender presentation at face value through the personal pronouns, and have indicated as much throughout. |
| 2. Line 240 - You have 'Many participants reported and isolating themselves from'. You need to edit this line for correctness and clarity. | 2. Thank you. We have restructured the sentence as recommended (see line 242).   *Adolescents in this study reported isolating themselves from others to prevent onward transmission of TB.* |
